# An optimized protocol for combined fluorescent lectin/immunohistochemistry to characterize tissue-specific glycan distribution in human or rodent tissues

**DOI:** 10.1016/j.xpro.2020.100237

**Published:** 2020-12-19

**Authors:** Ana Lúcia Rebelo, Paolo Contessotto, Kieran Joyce, Michelle Kilcoyne, Abhay Pandit

**Affiliations:** 1 CÚRAM SFI Research Centre for Medical Devices, National University of Ireland Galway, Galway H92 W2TY, Ireland; 2 School of Medicine, National University of Ireland Galway, Galway H91 TK33, Ireland; 3 Carbohydrate Signalling Group, Microbiology, School of Natural Sciences, National University of Ireland Galway, Galway H92 W2TY, Ireland

**Keywords:** Microscopy, Molecular Biology, Tissue Engineering, Glycobiology

## Abstract

Lectin histochemical analysis of tissues combined with immunohistochemistry is a valuable tool to characterize and correlate the spatial distribution of glycans with the presence of specific cell types or antigens of interest. The current protocol describes the application of monosaccharide motif specificity of lectin binding to glycan residues to different tissue types. In addition, we describe stereological methods to provide further quantification of the analyzed tissues.

For complete details on the use and execution of this protocol, please refer to Mohd Isa et al. (2018), Contessotto et al. (2020), and Samal et al. (2020).

## Highlights

• Periodate-pretreated bovine serum albumin as a blocking reagent for lectin staining/IHC
• Combined lectin histochemistry and immunohistochemistry staining procedure
• Co-localization of glycan distribution with expression of antigens of interest
• Quantification of fluorescence intensity and positively stained area fraction

## Graphical Abstract

**Figure:**
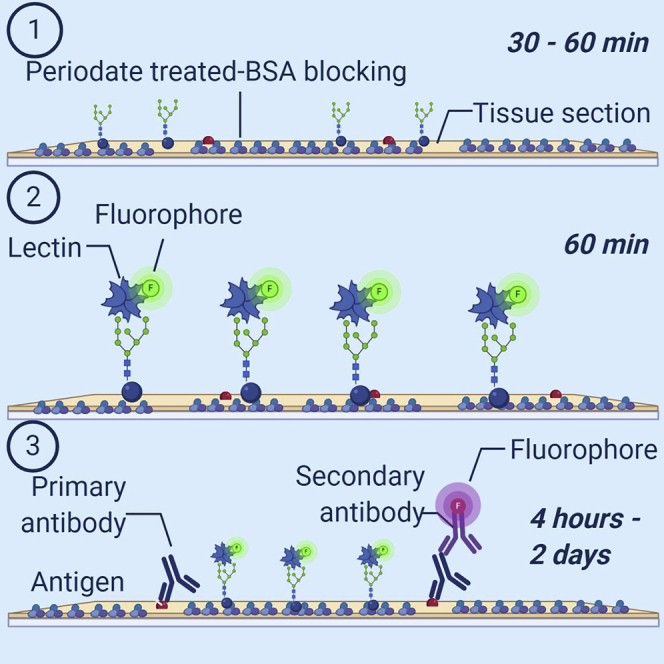

## Before you begin

### Experimental design considerations

1. Lectin/antibody staining with fluorescently tagged lectins or antibodies should be ideally performed in frozen (cryostat) tissue sections (either from human or animals), with a thickness that can vary between 5 and 10 μm to enable also high resolution imaging (maximum thickness of 10 μm would prevent super-imposition of histological features). In our experience, paraffin embedded tissue sections increase the non-specific binding of fluorescently labeled lectins, enhancing background staining. The use of permanently positively charged glass slides (SuperFrost Plus) is strongly recommended to avoid the detachment of the tissue sections during this procedure.
2. Throughout the whole staining procedure, tissue sections need to be protected from direct light exposure as fluorescently (e.g., FITC/TRITC-) labeled lectins quench more quickly than Alexa Fluor-labeled probes. Additionally, stained sections should be imaged within 4 days to ensure specific binding. Fluorescently labeled lectins should be stored according to the manufacturer’s instructions. Stock solutions (after reconstitution, in case the lectin is purchased as a powder) should be aliquoted and kept at −20°C for long-term storage, or at 4°C for up to 1 month. Avoid freeze-thawing cycles and always protect fluorescently labeled lectins from direct light exposure.
3. An optimization of lectin concentration and incubation time should be done to ensure the optimal concentration will be used in the following analyses (suggested concentrations for the titration: 5 μg/mL, 10 μg/mL, 15 μg/mL, 20 μg/mL). Please note that optimal concentrations will depend on the lectin as well as on the tissue used. The optimal concentration of lectin to be used is achieved when an increase in concentration above the optimal concentration does not increase the degree of staining of the tissue.
4. While the optimal concentration is being determined, the specific negative (inhibitory) control needs to be performed in parallel. Specifically, the lectin should be incubated for 30 min at room temperature with the sugar it is specific for (haptenic sugar) before staining the tissue section (selection of relevant lectins as well as their respective haptenic sugars is described in Table 1). This will enable the binding of the lectin to the sugar, resulting in the reduced binding to the tissue and subsequent decreased signal intensity, confirming the lectin specificity. It is also important to perform a positive control in parallel during the optimization step to guarantee that in case there is no labeling, it is not due to technical errors. Additionally, both negative and positive controls should be included in every batch of lectin labeling to ensure successful and specific labeling.

**Table 1:** Selection of most commonly used lectins, their binding specificity, and corresponding haptenic sugar for inhibitory control

Lectin	Binding specificity	Haptenic sugar
AAL (*Aleuria aurantia*)	Fuc-α(2,6)-GlcNAc	Fuc
AIA (*Artocarpus integrifolia*/ Jacalin)	Gal-β(1,3)-GalNAc	Gal
Con A (*Concanavalin* A)	α-Man, α-Glc	Man
DSA/DSL (*Datura stramonium*)	GlcNAc, GalNAc	GlcNAc/GalNAc
ECL/ECA (*Erythrina cristagalli*)	Gal-β(1,4)-GlcNAc	Lactose
GNL (*Galanthus nivalis*)	α-Man	Man
GSL I-B_4_ (*Griffonia* (*Bandeiraea) simplicifolia* I Isolectin B4)	Terminal α-Gal	Gal
MAA II (*Maackia amurensis* agglutinin II)	Neu5Ac-α(2,3)–Gal	Lactose
PNA (Peanut agglutinin)	Gal-β(1,3)–GalNAc	Gal
RCA I (*Ricinus communis* I)	Gal	Gal or lactose
SNA-I (*Sambucus nigra* agglutinin I)	Neu5Ac-α(2,6)–Gal/GalNAc	Lactose
UEA-I (*Ulex europaeus* agglutinin I)	α(1,2)-Fuc	Fuc
WGA (Wheat germ agglutinin)	GlcNAc-β(1,4)– GlcNAc	GlcNAc
WFA (*Wisteria floribunda* agglutinin)	GalNAc	GalNAc

Sugar abbreviations: Fuc – L-fucose; Gal – D-galactose; GalNAc – *N-*acetyl-D-galactosamine; Glc – glucose; GlcNAc – *N*-acetyl-D-glucosamine; Man – mannose; Neu5Ac – *N*-acetylneuraminic acid (sialic acid). Typically, haptenic sugar concentrations used are 100 mM but recommended concentrations can vary depending on manufacturer’s recommendations.
5. An optimization of primary antibody dilution and incubation conditions should also be carried out. The respective negative control should be done in parallel, where sections are incubated with a non-related primary antibody, to assess if there is non-specific binding of the secondary antibody to the tissue.
6. Bear in mind that phosphate-buffered saline (PBS) cannot be used throughout the whole procedure since phosphate ions bind to metal ions and sequester them from the lectin-binding sites (Brooks and Hall, 2012).

### Periodate treatment of bovine serum albumin

**Timing: 3 days**

***Note:*** Treatment of bovine serum albumin (BSA) is necessary to remove glycosylated contaminants (e.g., serum glycoproteins) that might promote lectin binding directly to BSA, interfering with the results of the assay (Glass, Briggs and Hnilica, 1981). It is recommended to use 99% purity BSA or greater to reduce any potential contaminants present as well.

7. Prepare 10 mM periodic acid in 0.1 M sodium acetate, at pH 4.5 (prepare fresh).

**CRITICAL:** Periodic acid is an oxidizing agent and a severe respiratory tract irritant. Therefore, while handling periodic acid, lab coat, gloves, powder particulate face mask, and safety goggles should be worn. Preparation of periodic acid solution as well as BSA dissolution should be done with caution in a fume hood, and it is crucial that the pH is kept at 4.5.

***Note:*** pH should be adjusted using glacial acetic acid and NaOH, if needed.

8. Dissolve high purity (99% purity or greater) BSA (Sigma, A7638) in freshly made periodic acid solution (recommended concentration: 5% BSA in freshly prepared periodic acid solution).
9. Incubate at room temperature (RT) (~18°C–23°C) for 6 h under mild stirring using a magnetic stirrer on a plate.

**CRITICAL:** Always visually inspect the sample to ensure that the BSA is completely dissolved. Do not vortex the solution as it creates bubbles.

10. Dialyze solution against deionized water for 48 h in a 5 liter beaker with stirring (Figure 1).

**Figure 1:**
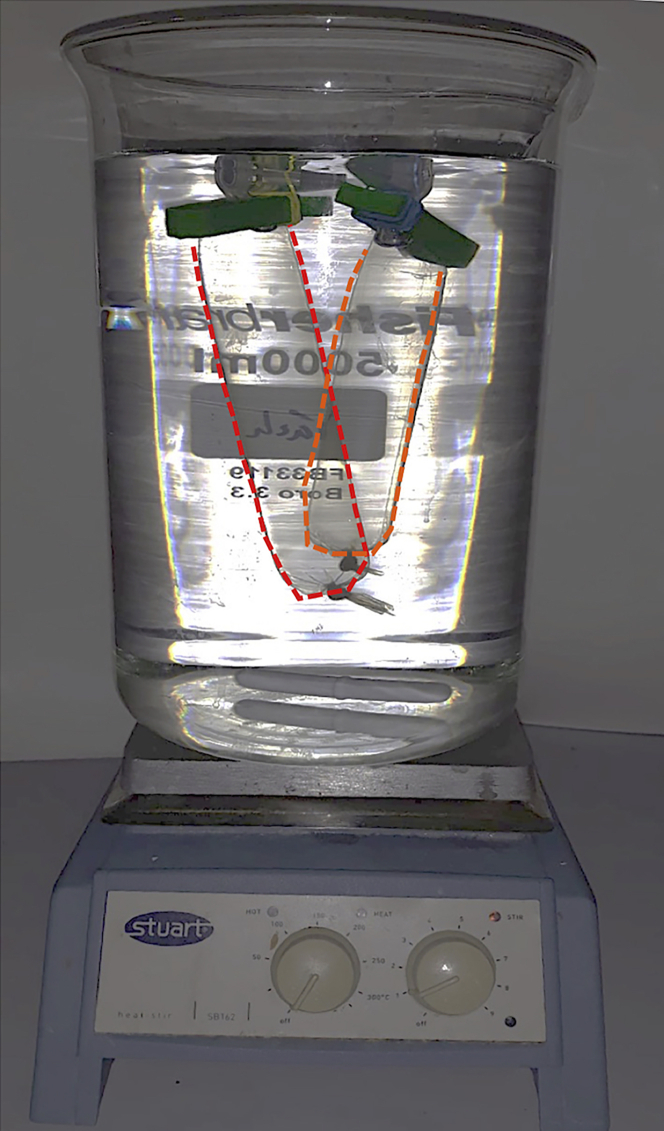
Experimental setup for dialysis of periodate-treated BSA (edges of dialysis membranes are dashed)
11. Keep dialysis set up at 4°C and change the water a total of four times (recommended to do it 3 h, 12 h, 24 h, and 36 h after starting the dialysis).
12. Freeze the solution at −80°C overnight (~16 h). It is recommended to freeze in aliquots of 20 mL inside 50 mL centrifuge tubes at an angle to facilitate the freezing of the inner core of the aliquots and to improve lyophilization.
13. Lyophilize it on the next day in a freeze drier until completely dry (for a volume of 20 mL it should take 2–3 days).
14. Store lyophilized periodate-treated BSA at 4°C in a sealed container (to avoid moisture). The color of the final powder should be between ivory white and white, and the texture should be fluffy (Figure 2).

**Figure 2:**
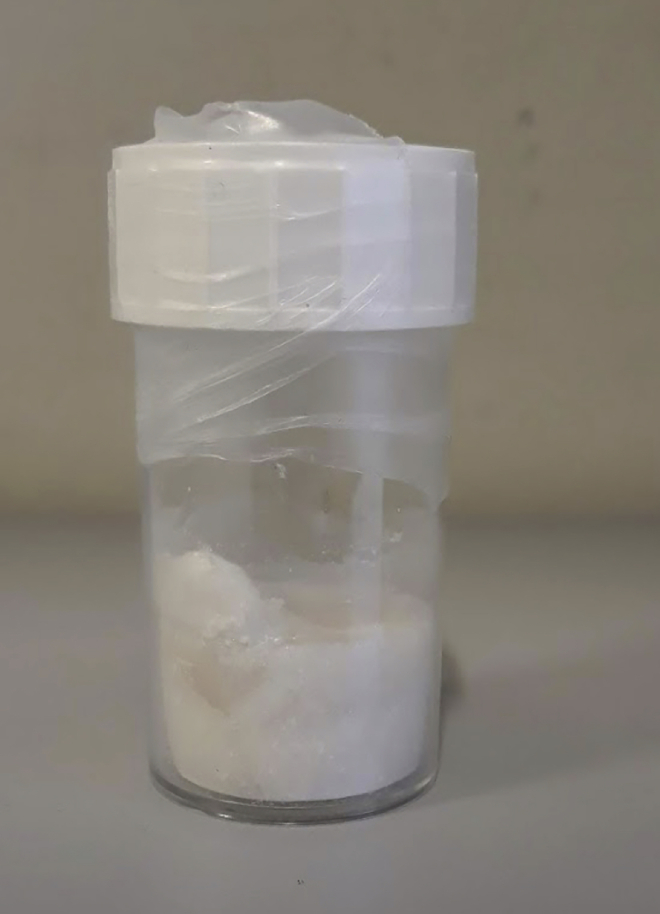
Final appearance of periodate-treated BSA Its color can range from ivory white to white, and it usually dries with a fluffy texture. If the resulting treated BSA is pink, orange, or brown, then the BSA is oxidized, and should be discarded as there will be solubility issues.

## Key resources table

**Table:** 

REAGENT or RESOURCE	SOURCE	IDENTIFIER
**Chemicals, peptides, and recombinant proteins**		

High purity (>99%) bovine serum albumin	Sigma-Aldrich	A7638
Periodic acid	Sigma-Aldrich	375810
Sodium acetate (CH_3_COONa)	Sigma-Aldrich	S5636
Trizma hydrochloride (TRIS-HCl)	Sigma-Aldrich	T5941
Sodium chloride (NaCl)	Sigma-Aldrich	S7653
Calcium chloride (CaCl_2_)	Sigma-Aldrich	746495
Magnesium chloride (MgCl_2_)	Sigma-Aldrich	M8266
Hydrochloric acid (HCl)	Sigma-Aldrich	30721-M
Sodium hydroxide (NaOH)	Sigma-Aldrich	S8045
Paraformaldehyde	Sigma-Aldrich	158127
Triton™ X-100	Sigma-Aldrich	T9284
Tween® 20	Thermo Fisher Scientific	85113
D-Mannose	Sigma-Aldrich	M2069
*N-*Acetyl-D-glucosamine	Sigma-Aldrich	A3286
*N-*Acetyl-D-galactosamine	Sigma-Aldrich	A2795
D-Lactose	Sigma-Aldrich	61345
L-Fucose	Sigma-Aldrich	F2252
D-Galactose	Sigma-Aldrich	G0750
Ethanol	Several suppliers	depends on supplier
Sudan black B	VWR	97061-982
Hoechst 33342	Thermo Fisher Scientific	33342
Fluoromount™ aqueous mounting media	Sigma-Aldrich	F4680
ProLong™ Gold Antifade Mountant with DAPI	Thermo Fisher Scientific	P36935

**Antibodies**		

Fluorescently labeled lectins	Vector Labs, EY Laboratories, Elicityl, Sigma-Aldrich, Thermo Fisher Scientific, and others	(lectin-specific)
Primary antibodies	Depend on the antibodies used. Suggested suppliers: Abcam, Thermo Fisher Scientific, Sigma-Aldrich, Santa Cruz Biotechnology, Cell Signaling Technology, and others	(antibody-specific)
Secondary antibodies	Depend on the antibodies used. Suggested suppliers: Abcam, Thermo Fisher Scientific, Jackson ImmunoResearch, and others	(antibody-specific)

**Software and algorithms**		

FIJI	ImageJ	https://imagej.net/Fiji

**Others**		

Dialysis tubing cellulose membrane 14,000 Da MWCO	Sigma-Aldrich	D9402
50 mL centrifuge polypropylene tubes	Corning	430829
Superfrost Plus™ glass slides (25 × 75 mm)	Thermo Fisher Scientific	10149870
Menzel™ Microscope Coverslips (24 × 60 mm)	Thermo Fisher Scientific	15747592
Glass Coplin staining jars	Sigma-Aldrich (or other suppliers)	S5766
Freeze drier	Labconco (or other suppliers)	7752061
Benchtop stirrer hotplate	Several suppliers	depends on supplier
5 L glass beakers	Several suppliers	depends on supplier
PAP pen	Several suppliers	depends on supplier
Opaque StainTray slide staining system	Merck	Z670146
Fluorescence confocal microscope	Olympus	Fluoview FV1000

**Biological samples**		

Post-mortem human brain tissue	Parkinson’s UK Brain bank	PDC008
Human intervertebral disc tissue	n/a	n/a
Mouse intervertebral disc tissue	n/a	n/a
Rat cardiac tissue	Charles Rivers, UK	n/a

## Materials and equipment

***Alternatives:*** In this protocol, a fluorescence confocal microscope is used. However, any fluorescence microscope could be used to image the staining performed as long as it has the adequate filters/lasers needed to image the fluorescent tag on the lectins/antibodies.

**Table:** Tris buffered solution (1× TBS) preparation

Reagent	Final concentration (mM)	Amount
Tris-HCl or Tris base	20	3.152 g if using Tris-HCl 2.422 g if using Tris base
NaCl	100	5.840 g
CaCl_2_	1	0.111 g
MgCl_2_	1	0.095 g
Double distilled (dd) H_2_O	n/a	Add to 1 L
**Total**	**n/a**	**1 L**

**CRITICAL:** Presence of CaCl_2_ and MgCl_2_ is crucial for lectin function. Store autoclaved or filter-sterilized solution at RT (~18°C–23°C) for a maximum of 3 months.

**CRITICAL:** Adjust pH to 7.2 with concentrated HCl or 1 M NaOH, depending if Tris base or Tris-HCl are used initially, respectively. HCl is corrosive and irritant to skin and eyes, so adjustment of pH should be done inside a fume hood. While handling HCl, lab coat, gloves, powder particulate face mask, and safety goggles should be worn.

***Note:*** To prepare TBS-T, add 0.05% of either Triton™ X-100 or Tween® 20 to TBS. Whichever detergent is selected, it should be consistently used as these are not interchangeable. Tween® 20 is more soluble than Triton™ X-100, so the Triton™ X-100 solution should be gently mixed after addition of the detergent.

***Note:*** Do not use phosphate-buffered saline (PBS) in this protocol since phosphate ions bind to metal ions and sequester them from the lectin-binding sites (Brooks and Hall, 2012).

**Table:** 4% paraformaldehyde (PFA) solution preparation

Reagent	Final concentration	Amount
Paraformaldehyde	4%	40 g
1× TBS	n/a	Add to 1 L
**Total**	**n/a**	**1 L**

***Note:*** Paraformaldehyde is toxic, flammable, carcinogenic, corrosive, and irritant to skin, eyes, and lungs airways. Therefore, while handling PFA, lab coat, gloves, powder particulate face mask, and safety goggles should be worn. All steps should be carried out inside a fume hood. PFA waste should be disposed of safely, in a specific container.

**CRITICAL:** PFA should be dissolved in 800 mL of 1× TBS at 60°C with continuous stirring, inside a fume hood. Add concentrated NaOH until solution is clear and allow it to cool. Filter the solution, adjust pH to 6.9 with concentrated HCl and complete to 1 L with 1× TBS. The final 4% PFA solution can be kept for up to 1 month at 4°C or aliquoted and stored long term at −20°C.

## Step-by-step method details

### Lectin staining of fixed-frozen tissue sections

**Timing: 4 h**

In this section, lectin histochemistry of tissues is performed to investigate specifically the spatial distribution of glycans in the tissue, which can be eventually combined with antibody staining. Tissue-dependent modifications are outlined in the relevant steps.

***Note:*** This protocol is a combination and optimization of other methods previously published by (Brooks and Hall, 2012; Mohd Isa et al., 2018; Kilcoyne et al., 2019; Contessotto et al., 2020).

**CRITICAL:** Once the staining procedure starts (after the first wash and throughout the whole procedure), it is crucial to always keep the tissue wet and not allow it to dry.

1. Leave tissue frozen (cryostat) sections on a slide warmer at room temperature (~18°C–23°C) between 30 min to 1 h (depending on the size of the tissue) before starting the staining to enable evaporation of moisture and to improve tissue adhesion to the slide.
  a. If the tissue used was not fixed before being frozen, incubate sections with 4% PFA solution on the slide for 2 min (inside fume hood)
2. Circle/frame each tissue section with a PAP pen to avoid leakage of the lectin solution away from the tissue section. The PAP pen creates a hydrophobic barrier, allowing the use of less reagents.

***Note:*** If the hydrophobic barrier vanishes throughout the following steps, carefully dry the borders of the glass close to the tissue section and repeat this step.

3. Wash sections with TBS-T three times, for 3 min each at RT (~18°C–23°C) in a glass Coplin staining jar placed on a shaking platform at a low speed (60 – 80 rotations per minute (rpm)).
  a. Monitor the sections to be sure they do not detach from the slide. If this happens, check Troubleshooting 1.
4. Lay out the slides with the fixed-frozen sections horizontally, with the tissue section facing up, in an opaque (blackout) StainTray slide staining system.

***Alternatives:*** If a StainTray slide staining system is not available, the slides might be placed on glass rods in an opaque sandwich box lined with damp filter paper or other similar alternatives.

5. Block sections with 3% periodate-treated BSA in TBS, for 1 h at RT (~18°C–23°C).

***Note:*** Blocking solution should be prepared fresh just before the start of this procedure.

6. Wash sections with TBS three times, for 3 min each at RT (~18°C–23°C) in a glass Coplin staining jar, with gentle shaking.

**CRITICAL:** From this point, do not expose your samples to direct light for an extended period of time. It is suggested to carry out the remaining steps in darkness or under a red light to prevent the quenching of fluorescence.

**Figure 3:**
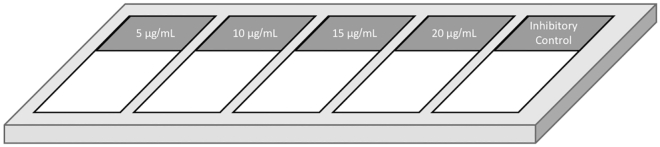
Suggested experimental design to perform at the beginning of the assay to optimize the lectin concentration to use and to confirm its specificity
  b. In parallel, perform haptenic sugar inhibition controls by pre- and co-incubating each lectin with 100 mM of the appropriate sugar in TBS-T at RT (~18°C–23°C) (Akimoto and Kawakami, 2014). See Table 1 for list of haptenic sugars to use with a selection of relevant lectins. Lectins should be prepared in the relevant sugar in TBS-T and pre-incubated for at least 30 min before adding to the tissue and continuing the sugar-lectin co-incubation on the tissue. A positive control should also be performed in parallel to ensure successful labeling. This could be done by using a tissue section that has been reported to display the glycan residues the lectin is specific to (Akimoto and Kawakami, 2014).
    ***Note:*** The specificity of a lectin is characterized by the sugar residue that inhibits the interaction between the lectin-binding site(s) and its respective glycan. This sugar (haptenic sugar) displays a competitive binding to the lectin-binding sites in relation to the carbohydrate motifs present in the tissue sample (Marks and Rodnight, 1978; Gerlach et al., 2012). Decreased binding in the presence of the haptenic sugar indicates the specificity of the lectin for the sugar. Complete inhibition (negative labeling) might also occur in some cases; however, this does not always happen and it is more common to expect a marked diminution in labeling intensity. No change in binding intensity indicates that lectin binding to the tissue was non-specific or mediated by a non-carbohydrate binding site on the lectin.

7. Incubate sections with fluorescently labeled lectins diluted in TBS-T for 1 h at RT (~18°C–23°C) in a dark chamber (e.g., StainTray slide staining system or an alternative opaque staining equipment).
  a. We recommend performing a titration experiment to identify the optimal concentration of lectin to be used, as mentioned above. See Table 1 for binding specificity of a selection of relevant lectins and Figure 3 for suggested experimental design.
    **CRITICAL:** Working solutions of lectins should be prepared fresh 30 min before each staining and discarded at the end of the experiment.
    ***Note:*** The fluorescent label on the lectins should be selected according to the lasers of fluorescence available in the microscope. However, fluorescein isothiocyanate (FITC) and tetramethylrhodamine isothiocyanate (TRITC) are the most commonly commercially available and used fluorescent tags for lectins.

8. Wash sections with TBS-T twice, for 3 min each, and then once with TBS∗ at RT (~18°C–23°C). We suggest performing drop washes while keeping the sections horizontal in the StainTray slide staining system (or alternative opaque staining equipment). The final wash in TBS without detergent avoids the retention of minimal bubbles on the tissue section.
  a. ∗If staining brain tissue: wash twice with TBS-T for 10 min each and twice with TBS for 10 min each. This is recommended for brain tissue due to its high content in lipids and in auto fluorescent lipofuscin, which increases the background signal. Longer and increased number of washes can help to reduce non-specific signal.

***Optional:*** Starting from this point, lectin histochemistry can be combined with immunohistochemistry. For more details, see next step “Antibody staining following lectin incubation.”

9. Counterstain either with Hoechst 33342, diluted 1:2,000 (final concentration at 0.01 mM) in TBS for 5 to 10 min, at RT (~18°C–23°C), or with ProLong™ Gold Antifade Mountant with DAPI (a single drop is sufficient for an approx. 0.5 cm^2^-sized tissue section).
10. If Hoechst 33342 is used, wash sections with TBS three times, for 3 min each∗ at RT (~18°C–23°C).
  a. ∗If staining brain tissue (or other tissues rich in auto fluorescent lipofuscin): after these washes, incubate with 0.1% Sudan black B solution in 70% ethanol (1 mg/mL) in a glass Coplin staining jar for 5 min at RT (~18°C–23°C). Wash then with TBS twice at RT (~18°C–23°C), for 4 min each.

***Note:*** Sudan black B solution can be re-used up to three times. Sudan black B binds to lipofuscin (granules rich in lipids, and abundant in the adult brain and other tissues), reducing/eliminating tissue auto fluorescence and background that usually arises from these granules (Oliveira et al., 2010).

11. After washing excess Hoechst 33342, apply one or two drops of Fluoromount™ aqueous mounting media and cover the slide with a glass coverslip (a single drop is sufficient for an approx. 0.5 cm^2^-sized tissue section). If ProLong™ Gold Antifade Mountant was used previously, this step would not be necessary.
12. Leave slides to cure overnight (~16 h) at RT (~18°C–23°C, in the dark) and seal around the edges of the coverslip with clear nail polish to prevent the coverslip from sliding and the mountant from drying out.
13. Keep slides at 4°C until imaging. We recommend imaging within 4 days for best results. For precious samples, stained slides can be stored at −20°C for up to 1 month.
14. Image stained slides by using a fluorescence microscope. Confocal microscopy enables the acquisition of images from several planes within the tissue section, in a Z-stack, providing a better resolution.

**CRITICAL:** Image stained slides within 4 days. For unexpected outcomes, check Troubleshooting 1.

### Antibody staining following lectin incubation

**Timing: up to 2 days**

In this step, immunohistochemistry can be carried out after lectin staining to complement the previous analysis. This procedure allows for the co-localization of glycan distribution with certain cell types or specific antigens present in the tissue. A general protocol is given, and tissue-dependent modifications are outlined in the appropriate steps.

**Pause Point:** Tissue sections already lectin stained can be kept overnight covered with TBS in a humidified chamber before starting the immunostaining procedure if the operator cannot continue immediately after performing the lectin histochemistry.

**CRITICAL:** The whole immunostaining step should be carried out in darkness or in a red-light room since the tissue was already incubated with fluorescently labeled lectins and it is crucial to prevent the quenching of the fluorescence. Additionally, the whole immunostaining procedure should be done by placing the slides in a humidified chamber to prevent them from drying.

***Note:*** A prior optimization for the immunostaining should be performed to detect the optimal primary antibody dilution and incubation conditions (time and temperature – usually the primary antibody should be incubated overnight at 4°C or for 2 h at RT). In addition, the usage of either TBS or TBS-T should be tested for the specific antibody to avoid interference with an optimal positive signal intensity (e.g., monoclonal vs polyclonal). The respective negative control should be done in parallel, where sections are incubated with a non-related or isotype control antibody, to assess if there is non-specific binding of the secondary antibody to the tissue. It may be appropriate to block with normal serum to avoid FC-mediated false-positive staining. However, this may affect previous lectin binding as serum contains glycoproteins, so appropriate controls should be employed during staining optimization.

**CRITICAL:** The whole immunostaining step should be carried out continuing to use TBS and not switching to PBS.

15. Keep slides in the StainTray slide staining system (or alternative opaque staining equipment) and block with 3% periodate-treated BSA in TBS, for 1 h at RT (~18°C–23°C).

***Note:*** This step (2^nd^ blocking) is not crucial and might be skipped. This will depend on antibody staining, as nuclear antibody stains may require serum-based blocking. While this may have an effect on previous lectin staining, this must be optimized by the end-user using appropriate controls to determine suitability of each counterstain, ensuring specificity and minimal off-target binding. Immunohistochemical counterstaining should be reconsidered if interference occurs.

16. Incubate sections with primary antibody at the concentration, time and temperature optimized previously (usually the primary antibody should be incubated overnight (~16 h) at 4°C or for 2 h at RT (~18°C–23°C), diluted in either TBS or TBS-T).
17. Wash sections twice with TBS-T for 10 min each and once with TBS for 10 min, at RT (~18°C–23°C). These should be drop washes while keeping the sections horizontal in the StainTray slide staining system (or alternative opaque staining equipment)∗.
  a. ∗If staining brain tissue (or other tissues with potential auto fluorescence): vertical washes are recommended using a glass Coplin staining jar.
18. Incubate sections with fluorescently labeled secondary antibody diluted in TBS (at optimized dilution) for 1 h at RT (~18°C–23°C).

**CRITICAL:** The secondary antibody and lectin used must be labeled with fluorophores with a narrow absorption wavelength without spectral overlap with each other. For instance, if the lectin is FITC-labeled (λ_ex_ = 488 nm, λ_em_ = 525 nm), then the secondary antibody cannot be Alexa Fluor 532 (λ_ex_ = 532 nm, λ_em_ = 554 nm) as they would emit at an overlapping wavelength spectrum, causing significant bleed through. A more appropriate combination would be FITC (λ_ex_ = 488 nm, λ_em_ = 525 nm) and Alexa Fluor 647 (λ_ex_ = 650 nm, λ_em_ = 671 nm), for example.

19. Wash sections with TBS three times, for 3 min each∗ at RT (~18°C–23°C). These should be drop washes while keeping the sections horizontal in the StainTray slide staining system (or alternative opaque staining equipment).
  a. ∗If staining brain/cardiac tissue (or other tissues rich in auto fluorescent lipofuscin): wash once with TBS-T for 10 min each and three times with TBS for 10 min each; vertical washes are recommended using a glass Coplin staining jar.
20. Counterstain either with Hoechst 33342, diluted 1:2,000 (final concentration at 0.01 mM) in TBS for 5 to 10 min at RT (~18°C–23°C) or with ProLong Gold Antifade Mountant with DAPI (single drop is sufficient for an approx. 0.5 cm^2^-sized tissue section).
21. If Hoechst 33342 is used, wash sections with TBS three times, for 3 min each∗
  a. ∗If staining brain tissue (or other tissues rich in auto fluorescent lipofuscin): after these washes, incubate with 0.1% Sudan black B solution in 70% ethanol (1 mg/mL) in a glass Coplin staining jar for 5 min at room temperature. Wash then with TBS twice, for 4 min each, at RT (~18°C–23°C).
22. After washing excess Hoechst 33342, apply one or two drops of Fluoromount™ aqueous mounting media and cover slide with a glass coverslip (a single drop is sufficient for an approx. 0.5 cm^2^-sized tissue section). If ProLong™ Gold Antifade Mountant was used previously, this step would not be necessary.
23. Leave slides to cure overnight (~16 h) at RT (~18°C–23°C, in the dark) and seal around the edges of the coverslip with clear nail polish to prevent the coverslip from sliding and the mountant from drying out.
24. Keep slides at 4°C until imaging. For precious samples, stained slides can be stored at −20°C for up to 1 month.
25. Image stained slides by using a fluorescence microscope. Confocal microscopy enables the acquisition of images from several planes within the tissue section, in a Z-stack, enhancing image resolution. Example of an expected outcome is described in Figure 5.

**Figure 4:**
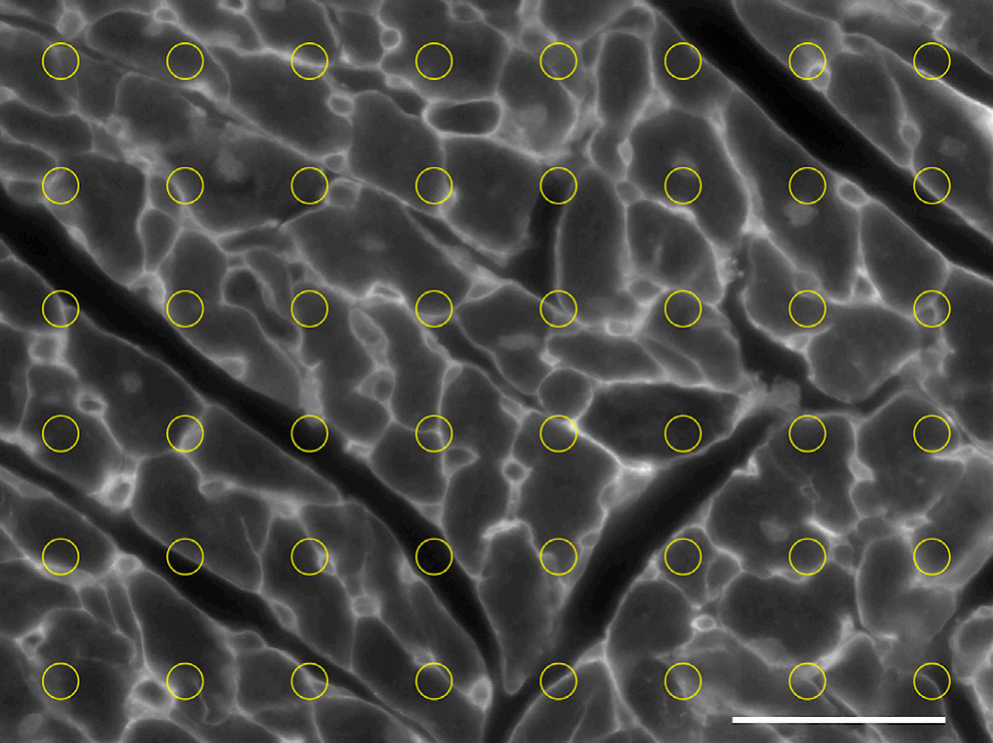
Example of a microarray grid to be superimposed on each image to perform an automated quantification of a sampled fluorescence intensity Scale bar, 50 μm.

**Figure 5:**
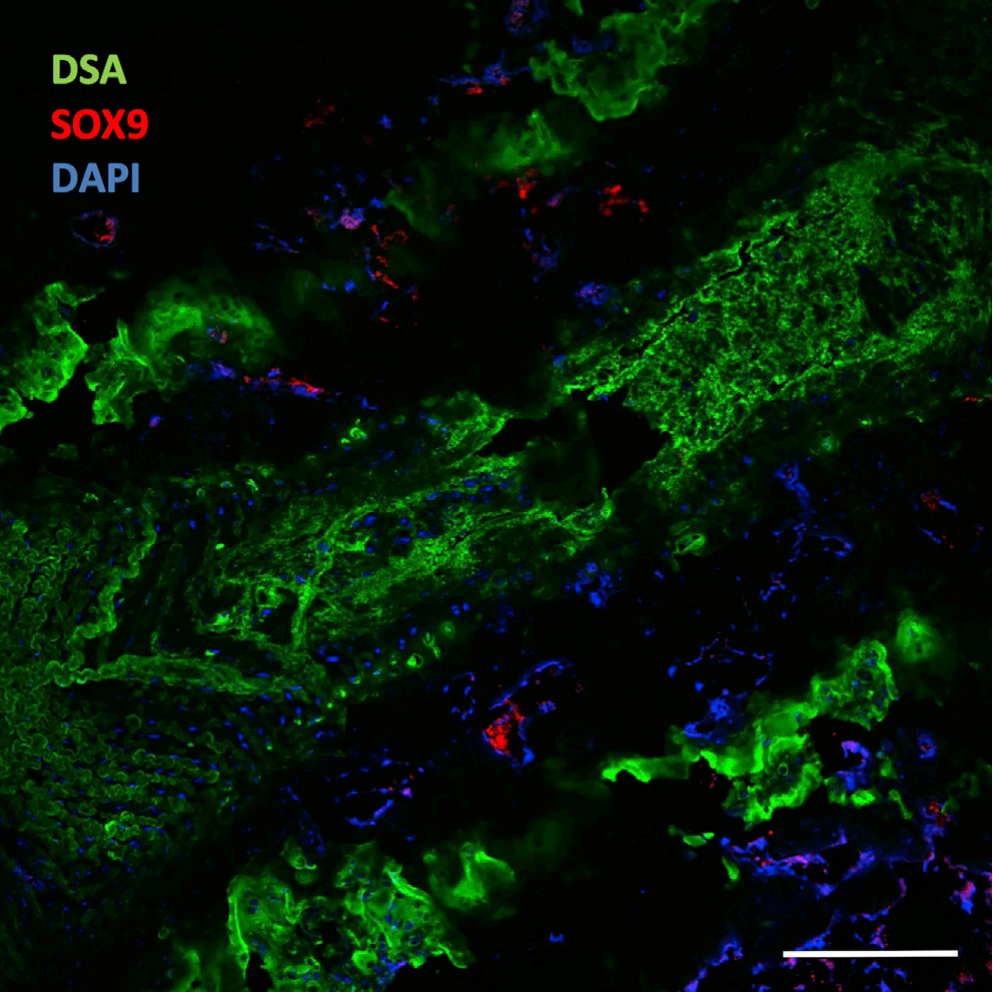
Example of an expected outcome from combined fluorescent lectin/immunohistochemistry using mouse intervertebral disc Scale bar, 200 μm. DSA, *Datura stramonium*; *SOX9*, *SRY-related box 9 transcription factor*; DAPI, 4′,6-diamidino-2-phenylindole.

**CRITICAL:** We recommend imaging within 3 days for best results.

## Expected outcomes

After performing the protocol for combined lectin and immunohistochemistry, it is possible to co-localize the expression of antigens of interest with glycan distribution. For example, in Figure 5, mouse intervertebral disc was stained with a marker for chondrocytic phenotype (*SOX9*, *SRY-related box 9 transcription factor*) and DSA lectin (*Datura stramonium*; specific for GlcNAc/GalNAc residues).

**Figure 6:**
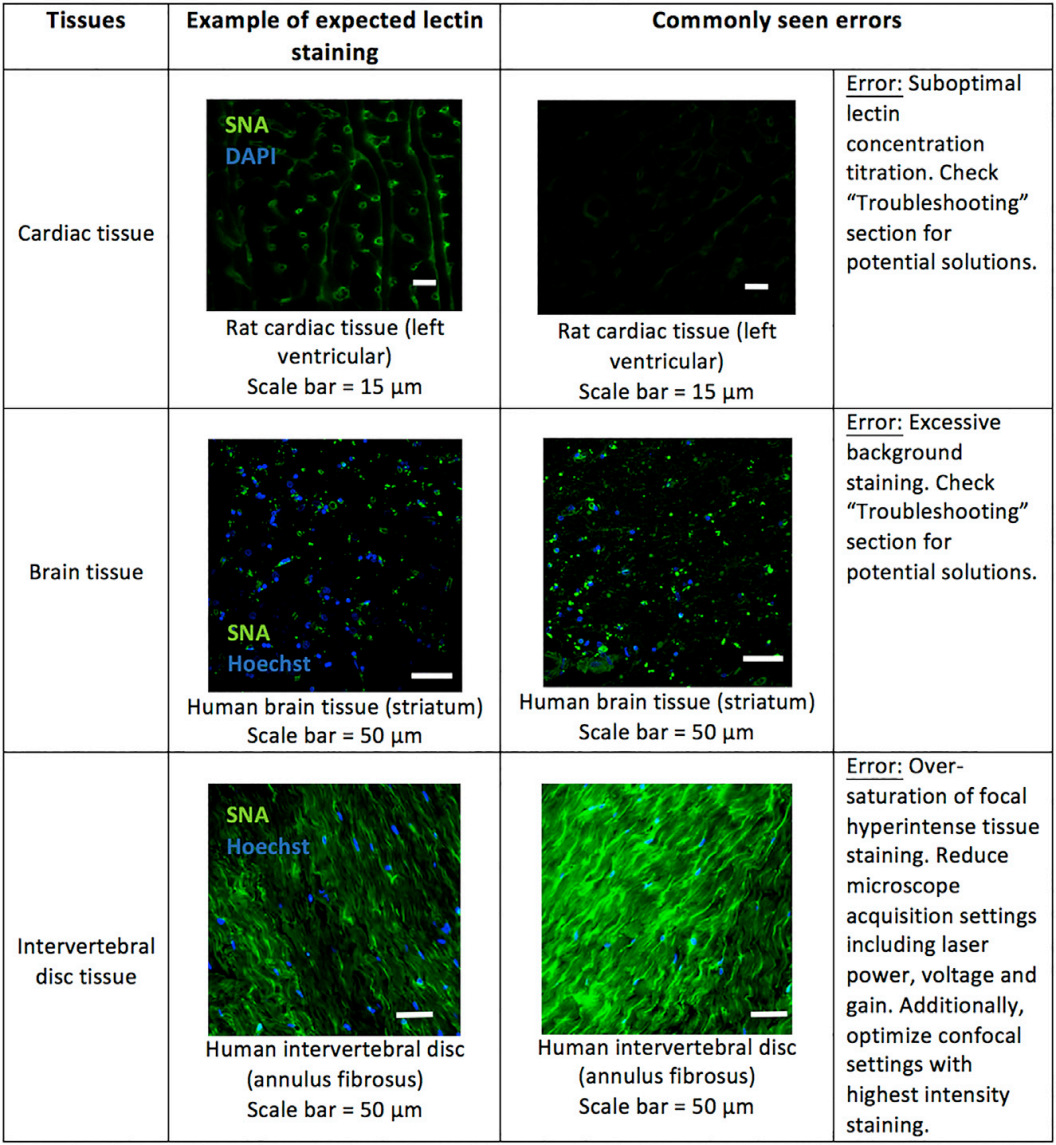
Examples of expected outcomes from lectin histochemistry using different types of tissues All tissues were stained with SNA I (*Sambucus nigra* agglutinin I), which is specific for Neu5Ac-α(2,6)–Gal/GalNAc residues. Examples of commonly seen errors are also provided, as well as potential solutions.

Since lectin histochemistry can be challenging and the outcomes can be very distinct depending on the type of tissue used, in Figure 6 there is a compilation of expected outcomes of SNA I (*Sambucus nigra* agglutinin I) staining on different tissue types (heart, brain, and intervertebral disc tissues). In the same figure, there are also examples of commonly seen errors, which will be helpful to unexperienced users to interpret their results and to predict what could be considered an expected result or a suboptimal outcome.

## Quantification and statistical analysis

### Quantifying area of tissue fluorescence with ImageJ/FIJI

Stereology quantification is one method that may be adopted to calculate the percentage (%) volume fraction of detectable lectin binding in each tissue sample. Lectin histochemical binding fluorescence should be obtained from eight to ten microscopic views of each slide with three technical and at least three biological replicates using FIJI/ ImageJ software version 1.51 (NIH, Bethesda, MD, USA). This is the recommended number of fields to acquire if magnification is 20×; however, if magnification used increases, then the number of fields acquired should increase as well. The use of z-stack image capture is recommended to reduce error in image focusing and perceived image intensity. Volume fraction (Vv) is calculated by quantifying the area fraction of the positively stained component divided by the total area of interest and converting into a percentage (%) as below:

$$Percentage\;Volume\;Fraction\;\left(\%Vv\right)=\frac{Area\;Fraction}{Total\;Area}\;x\;100\%$$

***Note:*** Prior to image processing, we recommend using .tif or .oib file types for future batch processing.

1. Open the micrograph to be analyzed in ImageJ/FIJI.
2. Convert the image to an 8-bit image (greyscale): ***Image* → *Type* → *8-bit.***
3. Set measurement scale by drawing a line over the scale bar, then: ***Analyze* → *Set scale.***
4. In the *Set Scale* window enter the actual distance in *Known Distance* box, confirm unit of measurement is appropriate and check *Global.*
5. Threshold the image using an automated routine: ***Process* → *Binary* → *Make Binary.***

***Note:*** This automated threshold includes only the area of fluorescent tissue.

6. Calculate the area of fluorescent tissue by surrounding the fluorescent tissue with the rectangular selection tool, and then: ***Analyze* → *Analyze particles.***
7. Enter the minimum particle size according to the magnification used and tissue stained, toggle Show Outlines, check Display Results, and click OK. Outline of the analyzed area will be drawn.
8. Calculate the entire area of the tissue by performing the threshold of the new image using manual setting: ***Image* → *Adjust* → *Threshold*** *(*Adjust sliders to include complete area of the tissue and click *Apply*)*.*
9. Surround the entire tissue with the rectangular selection tool, and then: ***Analyze* → *Analyze particles.***
10. Calculate %Vv as mentioned above.

***Alternatives:*** If the tissue section covers the entire image, follow steps 1 to 4 as stated above, and then employ a simple method of fluorescent area calculation as follows:

11. Select ***Analyze* → *Set Measurements,*** Check *Limit to Threshold*, then convert to binary image and select ***Analyze* → *Measure.***
12. Calculate %Vv as mentioned above

While these methods can accurately calculate area fraction of fluorescence, the authors recommend that if the tissue covers the entire background of the image, then only step one should be employed (assuming the entire area of the tissue = the area of the entire image). This way, batch processing can be performed in an entirely objective quantification protocol. Additionally, this whole protocol can be included as a Macro, which is a program that automates all these commands, allowing the quantification of the stained area to be done in one step.

### Quantifying intensity of tissue fluorescence with ImageJ/FIJI

Besides quantifying the area of tissue stained and subsequent volume fraction, another type of quantification that can be performed relates to the measurement of staining intensity in the tissue.

For that, a microarray grid should be used and the preliminary settings should be calibrated according to the distribution of the staining, which would be tissue-dependent as well.

13. Open the micrograph to be analyzed in ImageJ/FIJI.
14. Convert the image to an 8-bit image (grayscale): ***Image* → *Type* → *8-bit.***
15. Set measurement scale by drawing a line over the scale bar, then: ***Analyze* → *Set scale.***
16. In the *Set Scale* window enter the actual distance in *Known Distance* box, confirm unit of measurement is appropriate and check *Global.*
17. Apply the grid over the image: ***Analyze* → *Tools* → *Grid,*** and select Circles in Grid type. Define Area per point according to the tissue and staining used.
18. Calculate the mean intensity of fluorescent staining inside the grid points by selecting: ***Image* → *Overlay* → *To ROI Manager.***
19. In the ROI Manager, select GRID1 and check Measure. A new window will pop up, and the intensity measurement will be described as Mean Grey Value.

As an example, see Figure 4, where a circular-fitted microarray grid was set to quantify the fluorescence intensity staining of capillaries and cardiomyocytes membranes in light of the homogenous distribution of these morphological components in left ventricular cardiac tissue.

***Alternatives:*** If the “Grid” tool is not available, another similar plugin might be used after step 16.

20. Select ***Plugins* → *Microarray profile*** *(which is available online for free download as ImageJ jar)* **→*Change ROI*** *(Region of interest).* Set the shape according to the distribution of the region of interest (if the distribution of the ROI is homogenous, a circled one is suitable as a sampling unit) **→*Measure RT.***
21. A new window will pop up and the relative fluorescence intensity will be described as Mean Grey Value in a results table.

## Limitations

Although lectin histochemistry is an easy, quick, and straightforward protocol to establish across research groups focused on different fields, it has some limitations associated to it. The most important one would be that even though lectins can be used to detect carbohydrates in both glycoproteins and glycolipids, they can only discriminate between the identity of the actual carbohydrate residues present and not the nature of the glycosylated molecule. Additionally, lectins detect mainly terminal residues (Varki et al., 2009), and cannot elucidate the sequence of the entire oligosaccharide chain. For further information on full glycan structures present or the nature of the glycosylation molecule, the sample may be enriched for the membrane or extracellular matrix portions for further analyses on the glycans (e.g., lectin microarrays and liquid chromatography-tandem mass spectrometry (LC-ESI-MS/MS)). While highly informative in expert facilities, these investigative techniques remain highly challenging, with difficulty in interpretation and often require tissue-specific optimization.

Furthermore, although the use of fluorescently labeled lectins provides sharp and detailed images, and allows the co-staining with antibodies, it requires the imaging to be carried out within a short period of time post-staining, as their fluorescence fades within a few days.

The broad binding specificity of some lectins poses another significant limitation, since it might promote the binding of those lectins to structures that are not the ones of interest.

Moreover, the mostly qualitative (and only semi-quantitative) nature of this method presents some hurdles to an accurate quantification of the expression of each sugar residue in the tissue sample. This is due to the multivalent binding of lectins. Nonetheless, relevant stereological approaches can be further integrated in the analysis of the structures of interest (e.g., cellular membranes, extracellular matrix proteins). Quantification of lectin histochemistry images may be further validated using semi-quantitative western/lectin blotting techniques.

## Troubleshooting

### Problem 1

Sections detaching from charged glass slide (step 3).

### Potential solutions

When performing vertical washes (in glass Coplin staining jars), it is possible that tissue sections start to detach. To prevent this, numerous approaches can be taken into consideration:
  Solution 1: While cutting the sections in the cryostat and placing them onto the glass slides, they should be kept at room temperature for 30 min to 1 h to promote complete adherence of the tissue section to the glass.
  Solution 2: Slides such as APES (aminopropyltriethoxysilane) treated glass slides may be used (instead of SuperFrost™ Plus slides) to increase tissue adherence (https://theolb.readthedocs.io/en/latest/molecular-biology/apes-treatment-of-slides.html).
  Solution 3: Before starting the staining, enough time should be given for the slides to be at room temperature, to ensure that the sections are completely attached to the glass slides.
  Solution 4: If tissue is not already fixed, fix with PFA on slide to promote further adhesion.
  Solution 5: While performing the staining, gently replace the buffer between washes and, when using shaking, select a low speed.
  Solution 6: If the sections continue to detach, carry out all washes horizontally in a drop-wise fashion.

### Problem 2

High fluorescence signal is detected in sections that were incubated with the haptenic sugar (inhibitory controls) (step 14 or 25).

### Potential solution

If lectin binding is carbohydrate-mediated, by incubating a lectin with its haptenic sugar, the haptenic sugar will occupy the lectin-binding site and prevent the lectin from binding to the glycans in the tissue, leading to significantly decreased signal in those sections (complete abolition should not always be expected). Consult manufacturer’s instructions to confirm the most appropriate sugar to use. Additionally, it is worth noting that some lectins can only be inhibited by complex sugars and not monosaccharides, in which cases appropriately glycosylated glycoproteins should be employed (Brooks and Hall, 2012). If lectin binding is not significantly decreased in the presence of the haptenic sugar, lectin binding is either non-specific or mediated by the non-carbohydrate binding sites present in the lectin (Gerlach, et al., 2012). A different lectin with similar desired specificity should be selected in the latter case.

### Problem 3

High background staining/fluorescence signal (step 14 or 25).

### Potential solution

If a high background staining is detected, there are several steps that can be tried to reduce it. This can be due to several reasons:
  Reason 1: Insufficient blocking of non-specific binding sites, which can be overcome by increasing the concentration of periodate-treated BSA or by extending the incubation period of the blocking step.
  Reason 2: Excessive lectin concentration, incubation temperature, or incubation time. In these cases, it is advised to reduce the concentration of lectin and/or to incubate sections at 4°C instead of at room temperature and/or for a reduced period of time. Also, the lectin used could be potentially diluted in a buffer containing the blocking agent (e.g., 0.1% periodate-treated BSA in TBS-T or TBS). The use of other blocking agents (such as normal serum, which is frequently used in immunohistochemistry) is not appropriate as it possesses multiple glycosylated molecules, which might inhibit specific binding of lectins to the tissue or cause non-specific binding.
  Reason 3: Number and duration of washes are too short. These can be increased to ensure that any lectin that is non-specifically bound is removed.
  Reason 4: The tissue might have physiological structures/molecules that can cause auto fluorescence due to endogenous fluorophores (e.g., lipofuscin) (Croce and Bottiroli, 2014). To solve this problem, it is crucial to know the features and composition of the tissue and try to address them by finding a specific compound that can bind to these structures and reduce their auto fluorescence.

### Problem 4

No fluorescence signal is detected in the stained tissue sections (step 14 or 25).

### Potential solution

Lack of fluorescence signal in the samples might be caused by multiple factors:
  Factor 1: Not enough lectin is bound to glycans of interest, for which the recommended course of action would be to use a higher concentration of lectin, to incubate it during a longer period (e.g., 2 h or even 3 h) or to incubate it at a higher temperature (e.g., at 37°C).
  Factor 2: The lectin might have been improperly stored (e.g., kept at room temperature for long periods or in an environment not protected from light), which could lead to quenching of its signal or loss of its function. To ensure that the lectin has kept its function and labeling, a positive control should be run in parallel in every staining experiment. This positive control should be a tissue known to express the glycosylation motif specific to that lectin as characterized by the literature or based on previous in-house experiments.
  Factor 3: The specific glycosylation motif might not be present in the tissue sample. To confirm this, a positive control using a sample where this glycosylation motif has been reported to be found should always be run in parallel.
  Factor 4: The fixation procedure may modify the glycan structure that is necessary for recognition by the lectin. To solve this issue, a shorter fixation period or a different fixation method are recommended.
  Factor 5: TBS buffer might be contaminated with bacteria, which might alter its properties and compromise the staining and functions of the lectin. For this, it is advised to only use freshly prepared sterile-filtered or autoclaved TBS.

### Problem 5

The positive signal associated with the glycan distribution is not homogenous across the tissue section (step 14 or 25).

### Potential solution

Consider the histological nature of the region of interest where the lectin binding is localized. Suppose the region of interest has an extended and multi-layered morphology (e.g., artery), suitable confocal high magnification imaging needs to be implemented to detect the different layers of these structures. Then, only the relative areas of interest in ImageJ should be selected for fluorescence intensity measurement.

## Resource availability

### Lead contact

Further information and requests for resources and reagents should be directed to and will be fulfilled by the Lead Contact, Prof. Abhay Pandit (abhay.pandit@nuigalway.ie).

### Materials availability

This study did not generate new unique reagents.

### Data and code availability

This study did not generate/analyze any datasets/code.
